# Ontogeny of Thyroid Hormone Signaling in the Retina of Zebrafish: Effects of Thyroidal Status on Retinal Morphology, Cell Survival, and Color Preference

**DOI:** 10.3390/ijms252212215

**Published:** 2024-11-14

**Authors:** Iván Lazcano, Santiago M. Pech-Pool, María Fernanda Maldonado-Lira, Aurora Olvera, Veerle M. Darras, Aurea Orozco

**Affiliations:** 1 Instituto de Neurobiologia, Universidad Nacional Autonoma de México (UNAM), Campus Juriquilla, Boulevard Juriquilla 3001, Queretaro 76230, Mexico; 2 Facultad de Quimica, Universidad Autonoma de Queretaro, Centro Universitario, Queretaro 76010, Mexico; 3 Laboratory of Comparative Endocrinology, Biology Department, KU Leuven, 3000 Leuven, Belgium; 4 Escuela Nacional de Estudios Superiores, Unidad Juriquilla, Universidad Nacional Autonoma de México (UNAM), Campus Juriquilla, Queretaro 76230, Mexico

**Keywords:** thyroid hormones, retina development, zebrafish, deiodinases, thyroid hormone receptor

## Abstract

The retina is crucial for converting light into neuronal signals for visual perception. Understanding the retina’s structure, function, and development is essential for vision research. It is known that the thyroid hormone (TH) receptor type beta 2 (TRβ2) is a key element in the regulation of cone differentiation in the retina, but other elements of TH signaling, such as transporters and enzyme deiodinases, have also been implicated in retinal cell development and survival. In the present study, we investigated the expression profile of genes involved in TH signaling and analyzed the impact of thyroidal status on retinal morphology, opsin expression, cell death/proliferation profile, as well as color preference behavior during the early retina development of zebrafish larvae. mRNA expression analysis on dissected whole eyes revealed that TH signaling elements gradually increase during eye development, with *dio3b* being the component that shows the most dramatic change. Mutations generated by CRISPR/CAS9 in the *dio3b* gene, but not in the *thrb* gene, modifies the structure of the retina. Disruption in TH level reduces the cell number of the ganglion cell layer, increases cell death, and modifies color preference, emphasizing the critical importance of precise TH regulation by its signaling elements for optimal retinal development and function.

## 1. Introduction

The retina is a specialized neural tissue that plays a main role in converting light energy into an electrical neuronal signal that, in turn, is processed in the brain to create visual perception. Retinal morphology is well conserved among vertebrate species in terms of cell composition and constitution. Moreover, the retina contains five kinds of neuronal cells: photoreceptors (rods and cones) and amacrine, bipolar, horizontal, and retinal ganglion cells [1,2,3,4,5]. At the molecular level, several transcription factors that induce its development and differentiation in the early stages of development are also well conserved [6].

Thyroid hormones (THs) have been identified as crucial for the correct structure, function, and development of the vertebrate retina [7,8,9]. These hormones signal by binding to the nuclear TH receptors (TRs), which act as ligand-dependent transcription factors. TH receptor beta type 2 (TRβ2) regulates the differentiation of certain retinal cones that detect color in the retina of diverse vertebrates, from fish to mammals [10,11,12,13]. Mutations in the TRβ2 receptor are known to affect the development of long-wave-sensitive cones (L cones) in humans, retinal human organoids, and zebrafish [8,12,13,14,15]. Furthermore, other studies have shown that TH signaling impacts not only cone differentiation but also the survival of retinal cells [16,17]. While TRs mediate the final biological effects of THs, other components of the TH signaling pathway determine their nuclear availability. The cell membrane transporter monocarboxylate transporter 8 (MCT8) allows cellular TH influx and efflux, and its pivotal role in the correct development of retinal cones was recently demonstrated [18,19,20]. Other components, such as deiodinase (DIO) types 2 and 3, regulate TH activation/inactivation, respectively, determining nuclear T3 bioavailability [21]. Mutations in *DIO3* have been shown to decrease the survival of retinal cones in rodents [16,17], and *dio3b* knockdown disrupted eye and cone development in zebrafish [22]. Given that TH regulates all of these molecular components [13,23,24], it is reasonable to expect that TH-dependent retinal development will vary depending on thyroid status.

The zebrafish has been recognized as an excellent model for the study of retina development; it shares significant genetic similarity with humans, including the genes involved in retinal development and TH signaling physiology [19,25]. The transparency of zebrafish larvae facilitates direct observation of tissue and cell morphology as well as processes such as cone differentiation, cell proliferation, and cell death during eye development. In addition, several behavioral tests that evaluate retinal function in vivo have been well standardized [26,27,28].

To obtain more information about the dynamics of TH signaling and retinal development, we challenged the thyroidal status of zebrafish larvae during the period of retinal development, either increasing (exogenous T3 administration) or reducing with iopanoic acid (IOP) the T3 bioavailability. We analyzed the resulting effects upon the general anatomy of the retina, the expression of opsins, as well as cell death and proliferation status. We also conducted color preference paradigms to evaluate the behavioral effect of TH status on the in vivo color preference of larvae zebrafish. Our results show that even discrete fluctuations in TH levels can disrupt proper eye development and visual function.

## 2. Results

### 2.1. Expression Patterns of TH Signaling Genes During Retinal Development

**Figure 1:**
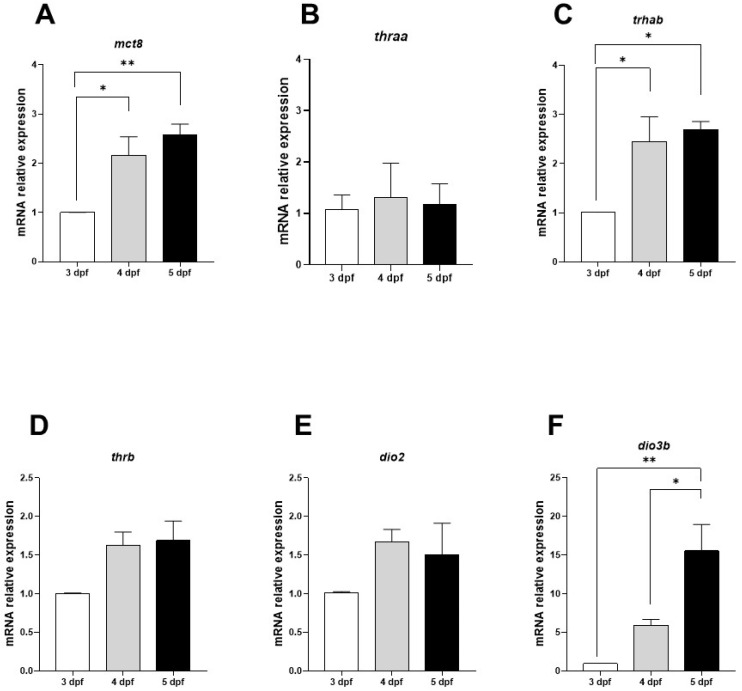
Retinal mRNA expression of *mct8*, *thraa*, *thrab*, *thrb*, *dio2*, and *dio3b* at 3, 4, and 5 dpf. (**A**–**F**) Each bar represents the mean fold change ± SEM. Data were obtained from four independent experiments, and each sample was analyzed in duplicate. Ribosomal 18S and actin mRNA were used as reference genes to apply the 2^−ΔΔCT^ formula. Statistical analyses were performed using one-way ANOVA and Tukey post hoc tests. Significant differences when comparing the control (3 dpf) to 4 and 5 dpf were indicated. * = *p* < 0.05, ** = *p* < 0.01.

*mct8* showed a significant increment from 3 to 4 dpf, *thrab* and *thrb* showed an increase from 3 to 4 dpf and remained constant at 5 dpf, and *thraa* did not show significant changes (Figure 1A–D). Regarding the deiodinase enzymes, *dio3b* showed the most dramatic changes (Figure 1F), increasing by 5- to 15-fold at 4 and 5 dpf compared to 3 dpf, respectively. On the other hand, *dio2* increased from 3 to 4 dpf, but this difference was not statistically different (Figure 1E). These results demonstrated the positive regulation of the thyroid signaling elements during early retinal development.

### 2.2. Retinal Phenotype of dio3b and thrb Crispants

**Figure 2:**
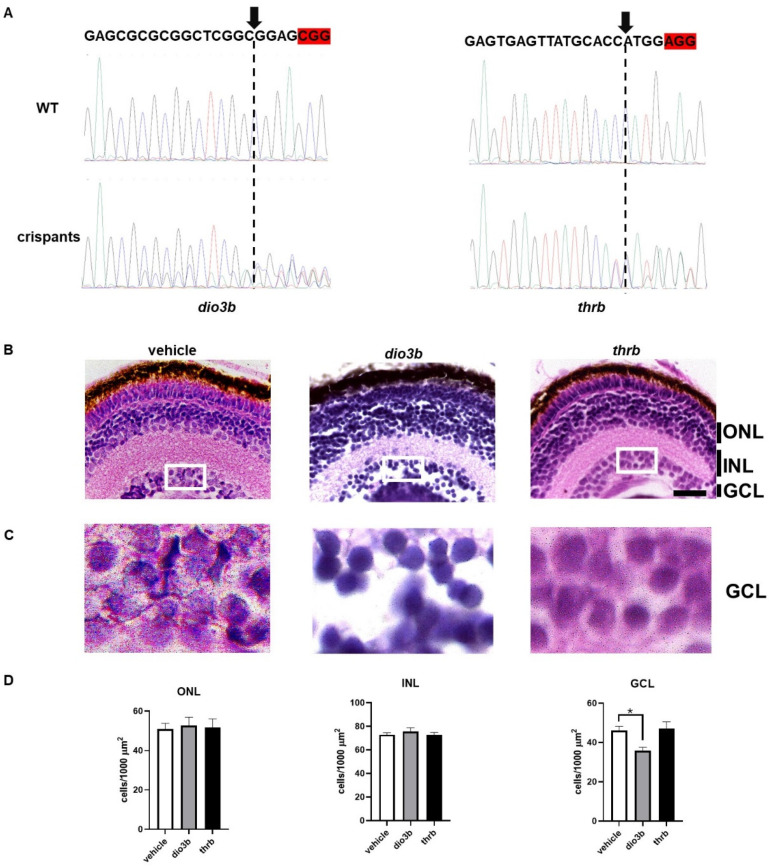
Comparison of *dio3b* and *thrb* crispants at retinal level. (**A**) Electropherograms from a fragment of the PCR amplicon sequence for *dio3b* and *thrb* of the vehicle-microinjected controls and the corresponding crispants. At the top, the sgRNA sequence is denoted, and the PAM sequence is highlighted in red. In the crispants, a decrease in the resolution of the electropherogram is evident around the canonical cutting site indicated with an arrow and a dotted line. (**B**) Representative histological cross-sections of retinas of vehicle-injected controls and *thrb* and *dio3* crispants (5–7 retinas per condition) stained with H&E. (**C**) Amplification of the GCL (insets in (**B**)). (**D**) Quantification of the number of cells/1000 µm^2^. ONL = outer nuclear layer, INL = inner nuclear layer, GCL = ganglion cell layer. Statistical analyses were performed using one-way ANOVA and Tukey post hoc tests. * = *p* < 0.05. Scale bar = 20 µm.

Several studies have analyzed the effects of *thrb* gene deletion on retinal cone differentiation in different vertebrates, but only a few studies have addressed the effect of the mutation of the *dio3* gene on retinal development. To analyze the consequences of the deletion of these genes, we used efficient CRISPR/Cas9 mutagenesis and compared the morphology of the early retina of crispant F0 animals for *dio3b* and *thrb*. F0 crispant larvae have proven to be an efficient and rapid screening tool for null phenotypes [29,30]. Using this approach, we found that *dio3b* crispants exhibited more pronounced alterations in the development of retinal layers, with a reduced cell number in the ganglion cell layer (GCL) compared to *thrb* crispants and vehicle-injected animals (Figure 2B–D). These results suggest that *dio3b* deletion has a greater impact on the overall development of retinal layers than that of *thrb*.

### 2.3. Thyroid Status Alters the General Morphology of the Zebrafish Larvae Retina

**Figure 3:**
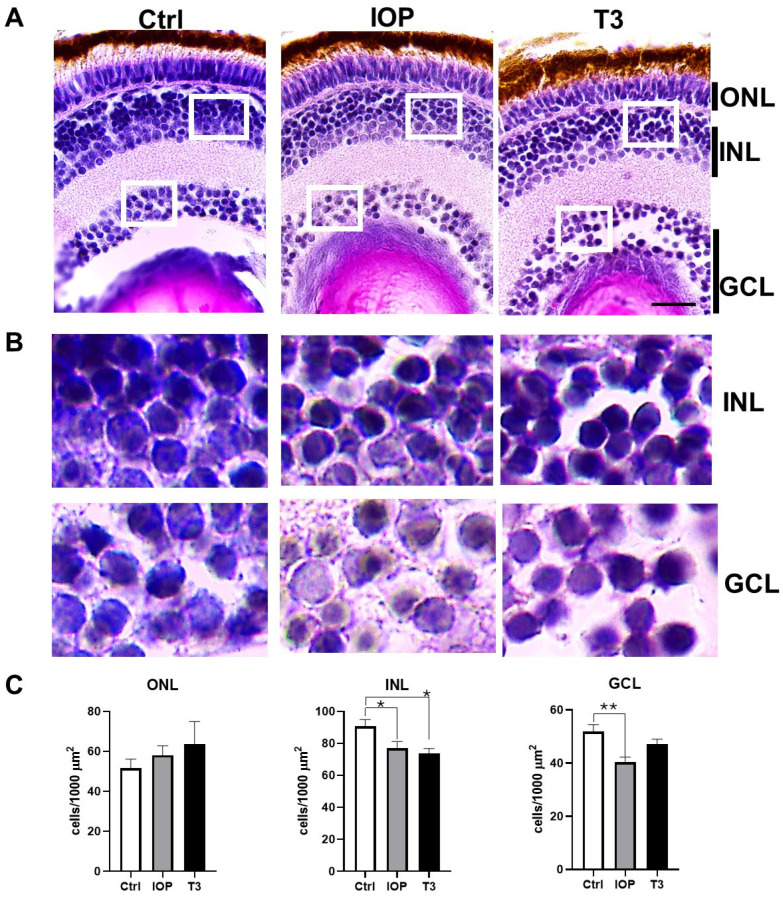
Thyroid status modifies the number of cells in the GCL and INL. (**A**) Representative histological retina cross-sections of control, IOP-treated, or T3-treated zebrafish larvae at 5 dpf stained with H&E. (**B**) Amplification of the INL and GCL (insets in (**A**)). (**C**) Quantification of the number of cells/1000 µm^2^ (n = 6) from the different experimental groups. Bars represent the mean ± SEM. Statistical analyses were performed using one-way ANOVA and Tukey post hoc tests. * = *p* < 0.05, ** = *p* < 0.01. ONL = outer nuclear layer, INL = inner nuclear layer, GCL = ganglion cell layer. Scale bar = 20 µm.

The zebrafish larvae treated with the antithyroid agent IOP or with exogenous T3 from 0 to 5 dpf showed evident changes in the structure of the retinal cell layers (Figure 3A). IOP exposure decreased the number of cells in the GCLs and in the inner nuclear layer (INL) (Figure 3B,C). On the contrary, exogenous T3 treatment only modified the cell number in the INL (Figure 3B,C). Although there was an increase in the cell number in the outer nuclear layer (ONL) in the larvae treated with T3, this difference was not significant.

### 2.4. IOP Decreases the Number of Axons in the Optic Nerve but Does Not Modify the Structure of the Optic Tectum

**Figure 4:**
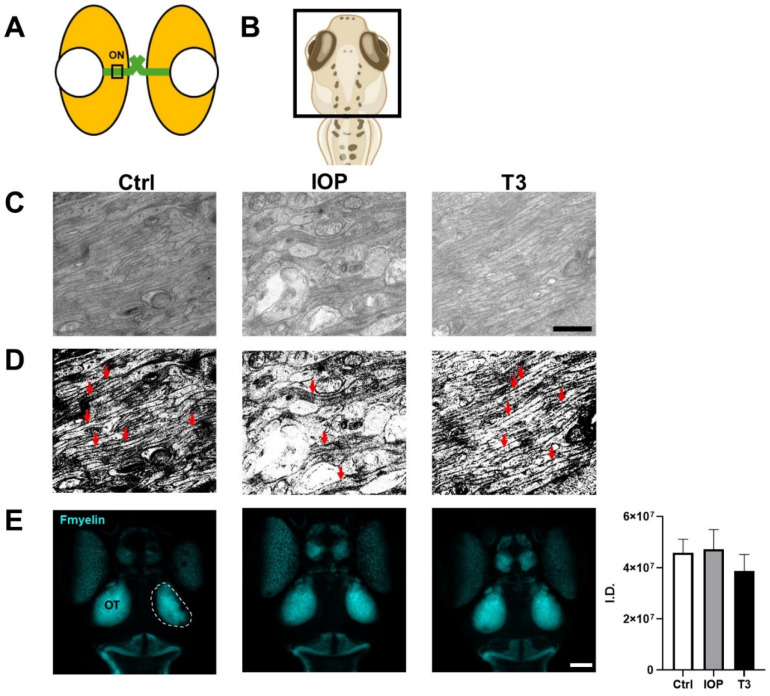
Thyroid status modifies the number of GCL axons but not the morphology of the optic tectum. (**A**) Illustrative representations of the zebrafish larvae eye indicating the optic nerve (ON) area photographed in (**C**,**D**). (**B**) Dorsal view representation to depict the Fmyelin stained area in (**E**). (**C**) Transmission electron micrographs of the optic nerve at 5 dpf and (**D**) their corresponding threshold. Axons crossing the optic nerve are fewer after IOP treatment, as indicated with the red arrows in (**D**). (**E**) Maximum intensity projections of the Fmyelin staining in the optic tectum (OT, circled in dotted line). Quantification from left and right OT is shown as integraded density (I.D.) from a Z-projection. Bars represent the mean ± SEM. n = 6 animals per group. Scale bar = 2 µm in (**C**) and 10 µm in (**E**).

In vertebrates, the cells of the GCL are those that form the optic nerve [27,31]. To show that the number of cells in the GCL is also reflected at the level of the optic nerve, we analyzed this structure using transmission electron microscopy in the different experimental groups. While the control animals and those treated with T3 showed a similar pattern of axons in the optic nerve, the animals treated with IOP showed a lower number of axons (Figure 4A), which correlates with the lower number of cells observed in the GCL under this treatment. In teleost fish, the first structure to receive information from the optic nerve is the optic tectum [32,33]. With the aim of analyzing whether our treatments could also be affecting the development of structures involved in visual processing, we analyzed the staining of myelin (Fmyelin) in the optic tectum in a whole mount preparation of control larvae and larvae treated with IOP or T3. In general, we did not find differences in the morphology and structure of the optic tectum (Figure 4B), which suggests that IOP and T3 treatments directly affect the retinal cells and the optic nerve but not the visual centers in the SNC.

### 2.5. Thyroid Status and the Expression of Zebrafish Opsins

**Figure 5:**
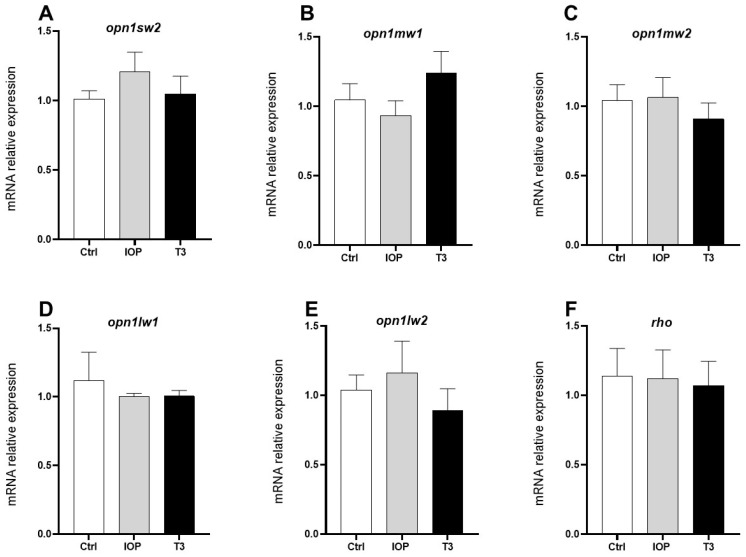
Effect of T3 and IOP treatments upon opsin mRNA expression in 5 dpf zebrafish larvae. (**A**–**F**) No significant statistical differences were observed when comparing the control (Ctrl) to T3-treated and IOP-treated groups. Each bar represents the mean fold change ± SEM from four independent experiments, and each sample was analyzed in duplicate. Ribosomal 18S and actin mRNA were used as reference genes to apply the 2^−ΔΔCT^ formula. Statistical analysis was performed using one-way ANOVA with multiple comparisons and Tukey post hoc tests.

One of the well-established TH effects is their regulation of the expression of the opsin 1 long wave sensitive (OPN1LW) gene, which detects the color red in humans, and the zebrafish homologue Opn1lw, which is duplicated in this species (Opn1lw1 and Opn1lw2) [12,13,15,34]. To verify if, in our paradigm, the expression of these opsins was regulated, we dissected the eyes of the IOP-treated and T3-treated 5 dpf zebrafish larvae and analyzed opsin expression by RT-PCR. Our results showed that, at that stage, neither treatment modified the expression of *opn1lw1* or *opn1lw2* (Figure 5D,E). Other opsin genes (*opn1sw2, opn1mw1, opn1mw2*, and *rho)* also did not change their expression when compared to the control group (Figure 5A–C,F).

### 2.6. IOP and T3 Treatments Differentially Modified Retinal Cell Death and Proliferation

**Figure 6:**
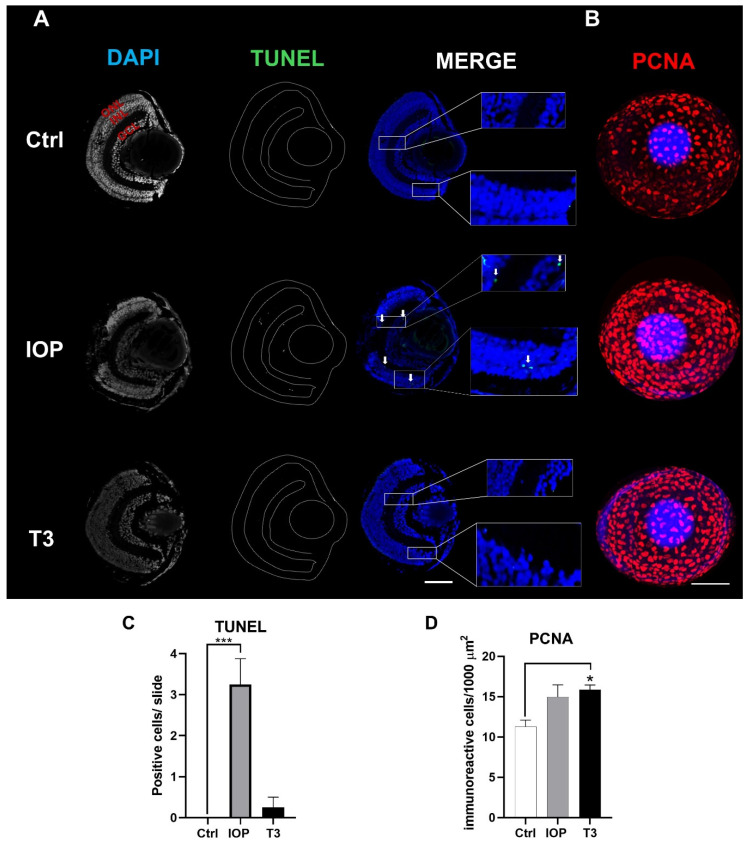
Cell death and cell proliferation after IOP and T3 treatments. (**A**) Microphotographs of sections of 5 dpf zebrafish retina showing apoptotic cells in retinal layers revealed by the TUNEL assay (green) and counterstaining with DAPI (blue). IOP induces an increase in apoptotic cells (white arrows) in the GCL and INL. (**B**) Confocal 3D reconstruction of a PCNA (red) whole mount retinal assay showing a significant increase in the proliferation profile in T3-treated larvae. (**C**) Quantification of apoptosis rate on the whole slide in the control (Ctrl), IOP, or T3 groups. (**D**) The quantification of whole-eye PCNA immunoreactivity in the control (Ctrl), IOP, or T3 groups. Bars represent the mean ± SEM. Scale bar = 50 µm. Statistical analysis was performed using one-way ANOVA with multiple comparisons and Tukey post hoc tests. * = *p* < 0.05, *** = *p* < 0.001.

Given that we found an evident decrease in the number of retinal GCL and INL cells after treatments with IOP and T3, we analyzed whether this could be due to an increase in retinal cell death. Using TUNEL assays, we found that only IOP treatment but not T3 treatment induced the appearance of apoptotic cells in the GCL and in the INL (Figure 6A,C). Because THs also regulate cell proliferation in different tissues, we took advantage of the transparency of zebrafish larvae to analyze the proliferation profile in a whole mount retinal assay using the cell proliferation marker PCNA. Surprisingly, both IOP and T3 treatments increased PCNA immunoreactivity in the zebrafish retina, although statistical significance was only observed in the group treated with T3 (Figure 6B,D). These results suggest that the thyroid status modifies both death and proliferation of neuronal cells in the retina.

### 2.7. Color Preference Is Modified After IOP and T3 Treatments

**Figure 7:**
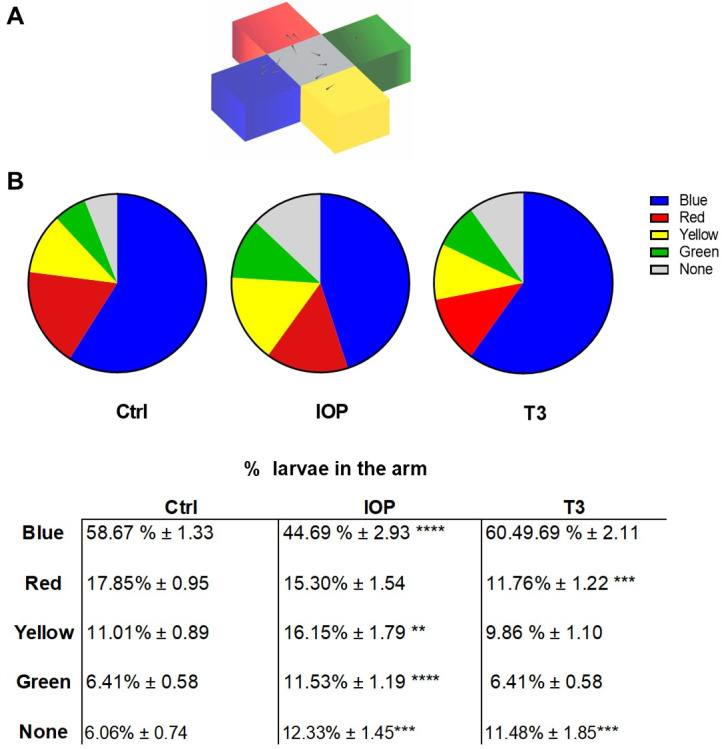
IOP and T3 modify the color preference paradigm. (**A**) Cross color maze and preference tests used in this study based on Park et al., 2016 [28]. (**B**) Color preference paradigm showing that IOP and T3 modify the pattern of color preference when compared to the control (ctrl) group (n ≥ 7 independent assays). Data are the mean ± SEM. ** = *p* < 0.01, *** = *p* < 0.001, **** = *p* < 0.0001.

To assess whether changes in the retinal cell number, cell death, and proliferation due to IOP and T3 treatments affect zebrafish larvae visual function in vivo, we employed a paradigm that evaluated the innate color preference at 5 dpf [27,28]. In all conditions, the zebrafish larvae preferred the blue arm (Figure 7B); however, the relative preference decreased for the IOP-treated larvae but not for the T3-treated larvae. Moreover, IOP treatment increased the yellow and green preference when compared to the control animals, while T3 only modified the red preference. Thus, IOP and T3 treatments modified the innate color preference.

## 3. Discussion

The role of THs in the correct development of the retina has been investigated for more than two decades. During this period, it has been demonstrated that THs participate in the correct development of the retina, particularly in cone differentiation. While the first studies demonstrated the fundamental role of the TRβ2 receptor in this process [11,12,13,15], later studies showed the role of DIO3 in cone survival [17], and recently, MCT8 was suggested as the main TH provider from the bloodstream to the retina [20]. Thus, TH signaling is key during retinal development.

We show the co-expression of TH signaling elements during early development in the eye of 3 to 5 dpf zebrafish larvae. It is important to note that most of the increases in the expression of the analyzed genes occur between 3 and 4 dpf. This coincides with important events of retinal maturation, such as the formation of circuits between the retinal cell layers, the innervation of the optic tectum, and the maturation of photoreceptors [12,26,27,35].

Interestingly, *dio3b* expression showed the highest increase. This could be linked to the fact that *dio3b* is expressed in most retinal cell layers during early development of mammals and zebrafish [17,22], while other TH signaling elements have a more restricted expression in this period. For example, during early retina development, TR expression is limited to the retinal cones in mouse, chicken, and zebrafish [10,11,20,36]. In contrast, *mct8* has only been detected in the developing retinal pigmented epithelium in mammals [20] and in multiple retinal layers in chicken [37]. For zebrafish, *Mct8* is present in the developing eye, but the specific cellular distribution has not yet been described [38]. The higher expression of *dio3b* could have a protective role against TH fluctuations that could occur during early development. There are several examples where DIO3 protects against TH overload, preventing premature response to T3 [39,40,41]. Therefore, the higher expression of *dio3b* could be preventing the premature differentiation of cones and/or protecting from cell death induced by T3 that has already been previously reported [34,41]. In agreement with earlier reports in zebrafish using morpholinos [22], knocking out the *dio3b* gene in the present study affected the structure of the GCL. Whether the observed reduction in neurons in the GCL was due to developmental defects or to cell loss due to degeneration remains to be investigated in more detail. Nevertheless, our results point to *dio3b* as a primary enzyme regulating the local concentration of THs in the developing retina in zebrafish.

Previous studies have shown that exogenous components that alter thyroid signaling, such as endocrine disruptors, exogenous T3, and antithyroid agents, can alter the structure of the retina [34,42,43]. Here, we used the last two to manipulate the thyroidal status. To our knowledge, this is the first study that analyzes the effects of the antithyroid agent IOP on retinal development, an agent that was proven to be effective in generating hypothyroidism in zebrafish larvae [44,45,46,47,48]. Our results demonstrated that early exposure of zebrafish larvae to either IOP or T3 during the first 5 dpf modifies the general structure of the retina, as shown by a decreased number of INL cells. In contrast, only IOP exposure additionally affected the cell number in the GCL, while that in the ONL did not show significant changes.

Earlier studies demonstrated that opsin genes detecting medium wavelengths can be regulated by alterations in TH homeostasis. For example, it has been shown that the expression of the *opn1mw2* gene is decreased in *mct8*-mutant zebrafish, suggesting that this gene family could be downregulated when there are insufficient levels of THs [19], while increasing concentrations of these hormones have been shown to downregulate the *opn1lw2* gene [34]. In the present study, we found no significant changes in the expression of the T3-responsive opsin genes at 5 dpf. The differences between previous reports and our results may be, at least in part, explained due to differences in the developmental stages analyzed (4 vs. 5 dpf) as well as to the concentration of T3 treatments (4 to 100 nM vs. 0.025 nM) [34]. We did, however, observe a trend of the *opn1mw1* gene being downregulated with IOP but upregulated with T3 as well as an opposite trend for *opn1lw2* expression between the two treatments. Therefore, it is certainly worthwhile to investigate this in more detail in additional samples that include earlier developmental stages.

To further analyze the observed effects upon cell layer numbers after IOP or T3 treatments, we studied the cell death profile in 5 dpf retinal sections. The increase in the number of apoptotic positive cells after IOP treatment as observed by TUNEL assays suggests that the decrease in GCL and INL cell number could be due to an increase in cell death. Although we did not find apoptotic positive cells after T3 treatments, we do not rule out that cell death occurred in an earlier developmental window, for example, at 3 or 4 dpf. The precise molecular mechanisms by which T3 and IOP induce cell death have not yet been clarified; zebrafish could be an advantageous model to study these mechanisms.

Retinal cell proliferation (PCNA) increased after IOP and T3 treatments, although statistical significance was only found with the latter. Given that retinal cell number decreased with both treatments, cell proliferation could be triggered as a regenerative mechanism. Muller glia cells have been shown to regenerate lost cells after damage [49,50,51]. Immune cells, such as microglial cells, may also invade the retina and proliferate as a response to retinal degeneration [52]. Another explanation could be that the treatments delayed the developmental cell proliferation programs, which naturally decrease as retinal cells differentiate [53,54]. Although the molecular mechanisms are still unclear, an imbalance in T3 homeostasis results in the alteration of retinal cell proliferation.

T3 fluctuations not only alter cell number, death, and proliferation but also impact color preference, indicating that these insults also modify behavioral parameters. These changes in color preference did not concur with variations in the expression of opsin-related genes, which could have occurred at earlier stages of retinal development. Given that we did not find changes in the number of cells in the ONL and in opsin gene expression after IOP and T3 treatments, the changes observed in color preference may, at least in part, be due to the modifications in the number of GCL cells. It is well known that, in addition to participating in the correct transmission of information from the photoreceptors to the brain, these cells also participate in the fine integration of neuronal signals that lead to color discrimination [55,56,57]. Moreover, the correct formation of INL and GCL is pivotal for the proper functioning of the visual system [58]. Our results show that IOP treatment not only decreased the number of cells in the GCL but also decreased the number of axons in the optic nerve. This suggests that the effect of IOP on color preference is due to its direct effect on the retina. Although more subtle changes may be present throughout the brain, we do not find any indication for major problems with the anatomy of the OT visualized with Fmyelin.

Clinical reports of patients with different TH-related diseases sometimes present visual impairments [7,8,14,59]. For example, mutations in TRβ2 result in a decreased red color perception [14], while patients with hypothyroidism show a decrease in blue color perception [59]. These observations arise the interest to elucidate the cellular and molecular mechanisms that lead to these conditions.

In evolutionary terms, the participation of TH in retinal development has surely influenced the adaptation of the visual systems of different vertebrate lineages. By controlling photoreceptor differentiation and other aspects of retinal development, THs could have enabled species to fine-tune their visual systems to best adapt to their environments. In situations where species are exposed to endocrine disruptors that act as antithyroid agents, retinal development and/or color discrimination is altered, compromising food selection or predator avoidance [43,60]. For all these, the zebrafish represents a valuable research model to not only experimentally explore the conserved TH involvement during visual system development but to also address possible ecological outputs.

## 4. Material and Methods

### 4.1. Nomenclature

The zebrafish abbreviations for genes and proteins were assigned according to “Zebrafish Nomenclature Conventions”, where “*aaa*” was used for zebrafish genes and their transcripts and “Aaa” was used for zebrafish proteins. “*AAA*” was used for mammal genes, and AAA was used for mammal proteins.

### 4.2. Zebrafish

Wild-type zebrafish embryos were obtained from reproductive pairing between adult males and females. Embryos were collected at the one-cell stage and maintained under standard conditions at 28 °C in E3 medium (5 mM of NaCl, 0.17 mM of KCl, 0.33 mM of CaCl_2_, and 0.33 mM of MgSO_4_ with methylene blue) under a 12:12 h light:dark cycle. All procedures involving animal subjects were conducted in strict compliance with the national and international guidelines for laboratory animal care and use as well as the approval of the Ethics Research Committee of the Instituto de Neurobiología at the Universidad Nacional Autónoma de México (Protocol Number: 142.A). Euthanasia was performed by inducing hypothermal shock through immersion in ice water (2–4 °C).

### 4.3. Zebrafish Eye Dissection

For ontogenetic experiments, zebrafish eyes were dissected from 3, 4, and 5 days post-fertilization (dpf) larvae, as previously described [61]. Briefly, we anesthetized the larvae by cold immersion until general signs of immobility were observed. The larvae were accurately decapitated using razor blades to ensure euthanasia, and both eyes were quickly removed using a stereomicroscope. Pools of ~40–50 pairs of eyes were collected, kept in Trizol reagent, and stored at −80 °C for RT-PCR experiments.

### 4.4. Iopanoic Acid (IOP) and T3 Treatments

Zebrafish embryos were initially treated with three concentrations of T3: 0.025 nM, 0.25 nM, and 2.5 nM. The treatments with 0.25 and 2.5 nM T3 altered the overall larval movement at 5 dpf, rendering the color preference paradigm impractical. Therefore, all further experiments were conducted using 0.025 nM of T3.

Zebrafish embryos were kept in E3 medium (control) or exposed at 6 h post-fertilization (hpf) to T3 at 0.025 nM or IOP, an antithyroidal drug, at 5 μM. All treatment solutions as well as E3 medium alone or supplemented with T3 or IOP were changed every day for 4 days. At 5 dpf, the larvae were euthanized or submitted to a color preference paradigm (see below).

### 4.5. RNA Isolation and cDNA Synthesis

Total RNA was extracted from the zebrafish eyes with Trizol^®^ Reagent (Invitrogen, Carlsbad, CA, USA). cDNA was synthesized by retro-transcription with a RevertAid First Strand cDNA Synthesis Kit (Thermo Scientific, Vilnius, Lithiuania) from 500 ng of the total RNA. 

### 4.6. Quantitative PCR (qPCR)

All oligonucleotides used in the RT-PCR reactions were previously validated and published (Table 1). Primer specificity was confirmed by sequencing, gel electrophoresis, and melting curve analysis. mRNA was quantified in a QuantStudio 1 (QS1) Real-Time PCR System (Thermo Fisher SCIENTIFIC, Waltham, MA, USA). Each reaction contained 2 μL of cDNA (1:10 dilution), 3.3 μL of Maxima SYBR Green/ROX qPCR Master Mix (Thermo Scientific, Vilnius, Lithiuania), 2.06 μL of free RNAse water, and 0.32 μL of each reverse and forward oligonucleotide at 10 μM. The amplification conditions included an initial denaturation at 95 °C for 10 min, followed by 10 s at 95 °C, 10 s at 60 °C, and 10 s at 72 °C for 40 cycles. The melting curve consisted of 1 s at 95 °C, 1 s at 60 °C, and 1 s at 95 °C. The relative content of all mRNA transcripts was determined with the comparative threshold cycle (Ct) method and using the formula 2^−ΔΔCT^ [62] in which mRNA expression was relative to the geometric mean of 18 s and actin mRNA [63].

### 4.7. Hematoxylin and Eosin (H&E) Staining and Retinal Cell Layer Quantification

The zebrafish larvae (5 dpf) were fixed overnight in 4% PFA, paraffin embedded, sectioned at 5 µm, and stained with hematoxylin and eosin using standard protocols. Representative images from retina sections were taken with a light microscope from each experimental group. The optic nerve was used as a reference point for the quantification of the number of cells/1000 µm^2^. Microphotographs were manually counted in an area of approximately 1000 to 2000 square microns.

### 4.8. Transmission Electron Microscopy

The zebrafish larvae from 5 dpf were processed for inclusion in epoxy resin. They were fixed in 3% glutaraldehyde in cacodylate buffer for one hour, washed, and postfixed with 1% osmium tetraoxide in the same buffer for one hour and then washed again. The samples were then dehydrated in a series of ethanol solutions and flat-embedded in epoxy resin (Electron Microscopy Sciences Co., Cat. EMbed812, Hatfield, PA, USA). After polymerization, the tissue was cut to a thickness of 70 nm using an ultramicrotome (Model MTX; RMC Boeckeler Instruments Inc., Tucson, AZ, USA). Sections were cut using a diamond knife and placed on 300 mesh copper grids, contrasted with uranyl acetate and sodium citrate 0.2 M, washed with distilled water, and observed in the JEOL transmission electron microscope model JEM 1010. Images were obtained with a Gatan ORIUS model digital camera. We used a previous study to identify the optic nerve of the zebrafish larva under transmission electron microscopy visualization [64].

### 4.9. Crispant Generation

Microinjection. One-cell stage zebrafish embryos were injected with 1 nL of vehicle or working solution using 1.5 OD/1.12 ID thin-wall capillaries (World Precision Instruments) and a Pneumatic PicoPump (PV 820; World Precision Instruments, Sarasota, FL, USA).

The zebrafish *dio3b* and *thrb* gene sequences were obtained from Ensembl (www.ensembl.org). sgRNAs were designed and synthesized as previously reported [65] and are enlisted in Table 1. sgRNA for the *thrb* gene was designed to specifically knockdown the TRβ2 isoform. mRNA Cas9 and the respective sgRNA mix were prepared, and the zebrafish embryos were microinjected with a final volume of 1 nL, equivalent to 100 and 20 pg of Cas9 and sgRNA per embryo, respectively. Groups of approximately 50 fertilized eggs were injected with either sgRNA + mRNA of Cas9 guides or vehicle.

Crispant Verification. To verify that the zebrafish larvae contained the desired gene mutations, fragments of the *dio3b* and *thrb* genes that included the CRISPR/Cas9 target sites were amplified from individual 5 dpf microinjected larvae randomly picked from each experimental group (n = 50/group). Genomic DNA was extracted (KIT), equal concentrations (250 ng/μL) of gDNA from individual larvae were used for PCR amplification (Maxima SYBR Green/ROX qPCR Master Mix (Thermo Scientific, Vilnius, Lithiuania)), and the obtained reaction product was subsequently column-purified (DNA Clean & Concentrator™; Zymo Research, Irvine, CA, USA). Purified PCR amplicons were sequenced to verify the inserted gene mutation.

### 4.10. PCNA Staining

Immunostaining was performed according to [66] using the proliferating cell nuclear antigen (PCNA) as the primary antibody. Briefly, the zebrafish larvae (5 dpf) were fixed overnight with 4% PFA dehydrated in increasing gradients (25, 50, 75, and 100%) of methanol and stored at −20 °C. Afterwards, the larvae were rehydrated in decreasing gradients (100, 50, 75, 50, and 25%) of methanol, washed with phosphate buffered saline (PBS), permeated with 2 M of HCl for 20 min, and incubated in a 3% H_2_O_2_/0.5% KOH medium for 30 min to decrease pigmentation. To unmask antigens, the larvae were incubated in 100 mM, pH 6 of citrate buffer at 80 °C for 60 min. Subsequently, the larvae were washed with cold water for 5 min, immersed in 100% acetone for 30 min at −20 °C, and washed again with cold water for 5 min. Finally, the samples were incubated with a blocking solution (1% triton in PBS plus 0.1% bovine serum albumin [BSA]) for 60 min. The primary antibody anti-PCNA (1:100, Santa Cruz, Dallas, TX, USA) was incubated for two days at 4 °C. The secondary antibody (donkey anti-mouse IgG, 1:500, Invitrogen, Waltham, MA, USA) was incubated overnight at 4 °C. For micrograph eye captures, the zebrafish larvae were positioned laterally onto 1.5% low-melting-point agarose in concave slides. Fluorescence was detected with a Carl Zeiss-LSM 700 (Jena, Germany) confocal microscope, and micrographs were taken with ZEN software (SP5 FP3, black version) using a 20× objective and scanned three-dimensionally at 84 μm depth in a set Z-scan (0.66 pixels) at a resolution of 1024 × 1024 pixels. Post-image processing and analysis were performed using the Image J program from the Fiji package (1.54 g version).

### 4.11. Whole Mount Myelin Staining

Myelin visualization was performed using the Fluoromyelin (Fmyelin) staining kit (Invitrogen CAT: F34651, Waltham, MA, USA). The zebrafish larvae (5 dpf) were fixed overnight with PFA 4% and washed with PBS and stored at 4 °C. Afterwards, the larvae were depigmented with 3% H_2_O_2_/0.5% KOH medium for 30 min, washed with PBS, and incubated with 0.1% triton in PBS for 10 min. Finally, the larvae were incubated with Fmyelin (1:100) for 60 min at 25 °C. Fluorescence was acquired as previously mentioned but with a 10× objective and with 120 μm depth in a set Z-scan (0.66 pixels) at a resolution of 1024 × 1024 pixels. Post-image processing and analysis were performed using the Image J program from the Fiji package (1.54 g version). We manually select the left and right optic tectum to obtain the integrated density (I.D.).

### 4.12. TUNEL Assay

The zebrafish larvae were fixed in PFA as previously mentioned, paraffin embedded, and sectioned at 5 µm. At the time of the experiment, the tissue sections were deparaffinized (120 min at 60 °C) and rehydrated in decreasing gradients (100, 50, 75, 50, and 25%) of ethanol. Terminal deoxynucleotidyl transferase-mediated dUTP nick end-labeling (TUNEL) of nuclei was performed using a Click-iT™ Plus TUNEL Assay (Invitrogen, cat: C10617, Waltham, MA, USA) following the manufacturer’s protocol. The samples were counterstained with DAPI for 20 min and microphotographed with a Carl Zeiss-LSM 700 (Jena, Germany) confocal microscope and ZEN software (SP5 FP3, black version). The Image J program (1.54 g version) from the Fiji package (1.54 g version) was used for post-image processing.

### 4.13. Color Preference Paradigm

To test the color preference of the zebrafish larvae, we modified a previously reported paradigm [28]. Briefly, ten larvae (5 dpf) were placed in an acrylic plus maze containing E3 medium with four colored arms (blue, red, yellow, and green), each measuring 10 (W) × 35 (L) × 15 (H) mm. Behavioral movement patterns were recorded for 30 min using a digital camera. The number of larvae located in each arm of the maze was quantified every 3 min, and the percentage of the average position for each color and group was calculated.

### 4.14. Statistics

mRNA expression data were analyzed with one-way ANOVA and Tukey post hoc tests. The comparative references were the 3 dpf group for ontogenetic studies and the control group for IOP and T3 experiments. A *p* < 0.05 value was considered statistically significant.

## 5. Conclusions

Our study underscores the pivotal role of TH signaling in the proper development and functionality of the retina in zebrafish larvae. We highlight the significant expression and protective role of *dio3b* in the maturation and survival of retinal cells during early development. The findings reveal that a T3 imbalance leads to structural changes in the retina, predominantly affecting layer cell number, inducing apoptosis, and increasing cell proliferation. Notably, these alterations in retinal structure and cell dynamics correlate with changes in color preference behavior, suggesting that disruptions in TH signaling can impact visual perception. Our results highlight the phylogenetic importance of THs in regulating retinal development throughout vertebrate evolution as well as provide further evidence to the notion that THs have been a successful driving molecular factor in evolution.

## Figures and Tables

**Table 1:** Primers and templates.

Real-Time PCR Primers					
Gene Target	Sequence Identifiers	Forward Primer (5′-3′)	Reverse Primer (5′-3′)	Position	(pb)
*mct8*	NM_001258230	gttcgggaagatcggagacc	aacacggcacactgaggaat	exon 5–6	111
*thraa*	NM_131396.1	atggaaaacacagagcaggag	aggaacagagatgctcttgtc	exon 2–4	132
thrab	ENSDARG00000052654	ggatggaaataaggtgaatggaac	ggtagtgatatccggtagctttg	exon 3–5	210
thrb	ENSDART00000189391.1	gaaccacagccgttacacca	cactgcatctgagagaaatcc	exon 1	194
Dio2	NM_212789.4	gcagcgcatgttaaccacag	gttgtgggtcttaccgctga	exon 1–2	160
Dio3b	ENSDART00000131982.3	agggctccgcaggtgtg	aggaagtccagcaggcaggg	exon 1	106
Opn1sw2	NSDART00000011178.9	gggcaccaattacaagcaag	aggttacatgagaactgtgt	exon 1–5	1005
Opn1mw1	ENSDART00000002046.8	cagcccagcacaagaaactc	agagcaacctgacctccaagt	exon 1–2	190
Opn1mw2	ENSDART00000025241.5	tttttggctggtcccgataca	caggaacgcagaaatgacagc	exon 1–2	132
Opn1lw1	ENSDART00000065941.6	cccacactgcatctcgacaa	aaggtattccccatcactccaa	exon 6	63
Opn1lw2	ENSDART00000065940.6	agagggaagaactggactttcaga	ttcagaggagttttgcctacatatgt	exon 7	77
Rho	ENSDART00000027000.9	acttccgtttcggggagaac	gaaggactcgttgttgacac	exon 1	132
18s	BI897395.1	gaacgccacttgtccctct	gttggtggagcgatttgtct	no exon	118
Actin	NM_131031.2	tgaatcccaaagccaacagag	ccagagtccatcacaataccag	exon 3–4	139
gDNA Primers					
Gene target		Forward primer (5′-3′)	Reverse primer (5′-3′)		(pb)
*dio3b*	ENSDARG00000095767	cagtctgcgctgaagaacgc	gccgaagttgaggatcagcg		363
*thrb*	ENSDARG00000021163	gacatagcccatggtgtaag	ctttcttatgtggcccttgc		310
Templates for in vitro transcription of sgRNAs					
*dio3b*	taatacgactcactataGGGCGCGCGGCTCGGCGGAGgttttagagctagaa				
*trhb*	taatacgactcactataGGGTGAGTTATGCACCATGGgttttagagctagaa				
Generic	aaaagcaccgactcggcactttttcaagttgatagactagccttattttaacttgctatttctagctctaaaaac				

## Data Availability

Data is contained within the article.
